# Multi-UAV-Borne Surveillance Radar Trajectory Planning Method Based on Imitation Learning

**DOI:** 10.3390/s26092691

**Published:** 2026-04-26

**Authors:** Xuchao Gao, Mingqiang Li, Kai Guan, Jianjun Ge

**Affiliations:** Information Science Academy, China Electronics Technology Group Corporation, Beijing 100086, China; nossplz@163.com (X.G.); guankaer@163.com (K.G.); geradarnet@163.com (J.G.)

**Keywords:** multi-radar, trajectory planning, imitation learning, coverage modeling, signal interference countermeasures

## Abstract

To address the high computational complexity and insufficient real-time performance of traditional multi-radar trajectory planning methods in complex multi-platform sensing scenarios, this study proposes an imitation-learning-based trajectory planning method for multi-radar systems. The method features a trajectory policy neural network architecture based on multi-semantic information, and involves a training-data construction method with coverage rate as the optimization objective. The trajectory policy neural network is then trained via an imitation-learning algorithm with an auxiliary target. Simulation results show that the proposed method achieves an average coverage rate of 93.95%, and improves the single-step decision efficiency by a factor of 6.7 compared with heuristic-based trajectory optimization methods.

## 1. Introduction

Radars are now widely applied in many fields, including air traffic control, weather monitoring and prediction, maritime control and aerial industries [1]. Radar now plays an increasingly important role in the target tracking field [2]; as vital sensors for acquiring situational information, radar systems face severe challenges to their survivability and mission effectiveness in complex and highly challenging signal interference environments. The traditional operational mode relies on a single large early-warning aircraft or centralized radar platform, and due to its prominent target characteristics and concentrated system structure, the traditional mode is highly vulnerable to system failure or even complete paralysis under electromagnetic interference, signal suppression, or high-density interference scenarios. These countermeasures make it difficult to meet the operational requirements for sustained and stable perception in high-interference environments.

In recent years, with the rapid development of unmanned aerial vehicles (UAVs), communication technology, and distributed cooperative control theory, distributed multi-radar systems have gradually become a research hotspot. Recent developments in complex operational scenarios require that modern radars function as a radar network to overcome information uncertainties and limitations [3]. By distributing sensing, processing, and decision-making capabilities across multiple low-cost, cooperative unmanned platforms, multi-radar systems offer significant advantages such as target dispersion, high redundancy, and strong system survivability [4]. These systems show greater survivability, flexibility, and environmental adaptability in complex and highly dynamic electromagnetic interference environments. However, the operational effectiveness of multi-radar systems depends significantly on the trajectory planning and cooperative maneuvering capabilities of multi-radar members in dynamic environments. One of the important challenges in this field is how to achieve effective evasion of external interference sources while satisfying radar detection performance constraints.

To achieve efficient online trajectory planning for multi-radar systems in signal interference environments, this study proposes an imitation-learning-based trajectory planning method for multi-radar systems. First, a trajectory policy representation neural network architecture based on multi-semantic information fusion is designed, unifying the encoding of regional coverage status, target motion information, and radar own maneuvering state. Second, a training-data construction method with coverage rate as the optimization objective is proposed. By establishing a mathematical optimization model for trajectory planning and solving it offline, an expert demonstration dataset in the form of state–action pairs is constructed. Finally, an imitation-learning algorithm oriented toward coverage tasks is proposed to train the trajectory policy neural network. The network enables the policy model to rapidly infer and output heading control commands during the online phase, and significantly improves the real-time performance of trajectory planning while maintaining coverage performance. Simulation results demonstrate that the proposed method achieves an average coverage rate of 93.95%, and achieves a 6.7-fold improvement in single-step decision efficiency compared with heuristic-based trajectory optimization methods.

The remainder of this manuscript is organized as follows. Section 2 reviews related work; Section 3 presents the system model and problem formulation; Section 4 describes the proposed imitation-learning-based trajectory planning method; Section 5 presents simulation experiments and results; Section 6 provides discussion; and Section 7 concludes the study.

## 2. Current Research Status

Extensive research has been conducted on multi-radar system trajectory planning, with most existing studies focusing on mathematical modeling for optimal detection capability in interference-free environments. However, research focusing on signal interference scenarios, particularly active signal interference environments, remains relatively limited. For example, Yan et al. designed a UAV path planning approach considering attenuated regional coverage [5]; Lu et al. improved the multi-target tracking capability of airborne radar systems through path planning [6]; Duan et al. solved the path planning problem for multiple reconnaissance-strike integrated UAVs [7]; and Zhang et al. investigated path planning for UAV area coverage reconnaissance missions in interference-free conditions [8].

Regarding algorithms for solving trajectory planning models, existing research mostly adopts heuristic algorithms [9] or deep reinforcement learning algorithms. Heuristic methods have the advantages of intuitive modeling and ease of implementation, enabling the acquisition of feasible solutions or even near-optimal solutions to a certain extent. However, these algorithms generally suffer from high computational complexity, insufficient real-time performance, and limited adaptability to environmental changes. Especially in high-dimensional, highly challenging, and dynamically evolving signal interference scenarios, they struggle to meet the demands for rapid online decision-making. Among these, Zhang et al. proposed the novel secretary bird optimization algorithm (NSBOA) to optimize UAV paths [10]; Jiang et al. adopted the kite algorithm to design UAV obstacle avoidance path strategies in 3D space [11]; Wei et al. used the sparrow search algorithm to study trajectory planning in 3D space [12]; Liu et al. adopted the particle swarm optimization (PSO) algorithm to plan routes for maneuvering reconnaissance platforms [13]; Besada-Portas et al. applied genetic algorithms to multi-UAV path planning [14]; Shi et al. used particle swarm optimization and cyclic minimization methods to optimize routes under multi-target tracking [15]; Zhang et al. used a hybrid stochastic simulation and genetic algorithm to optimize the airborne radar network problem [16]; and Yuan et al. used the diffusion model to solve radar selection problem [17].

In recent years, reinforcement learning is able to learn optimal strategies through interaction with the environment without requiring precise modeling. It has become an important method for multi-UAV trajectory planning [18]. For instance, Tang et al. researched UAV autonomous trajectory planning based on prediction information in crowded unknown dynamic environments [19]; Li et al. studied autonomous vehicle trajectory planning in constrained spaces [20]; Xu et al. used the deep Q-network (DQN) method to study trajectory planning in a dynamic environment with complex 3D low-altitude terrain [21]; Gao et al. employed the deep deterministic policy gradient (DDPG) method to propose a trajectory planning method that avoids actual terrain [22]; Xu et al. implemented proximal policy gradient (PPO) to solve the path planning problem for UAV swarms in Global Positioning System (GPS) and communication denial environments [23]; and Wang et al. used multi-agent deep deterministic policy gradient (MADDPG) to study the UAV swarm pursuit decision-making problem in air combat environments [24]. Although reinforcement learning methods have strong potential in complex environmental perception and autonomous strategy learning, their training process typically relies on extensive online interaction. This leads to problems such as high sample acquisition costs, long training cycles, and insufficient stability. Consequently, it limits their engineering application in multi-radar trajectory planning tasks with highly demanding and strong real-time requirements. How to improve the training efficiency of trajectory policy networks has therefore become an urgent problem to be solved.

In this work, heuristic trajectory optimization methods are selected as the primary comparison baseline for two reasons. First, heuristic solvers remain one of the most widely adopted solution paradigms for multi-radar or multi-UAV trajectory planning problems, especially in scenarios involving nonlinear constraints and high-dimensional decision spaces. Second, the proposed imitation-learning framework is trained using expert demonstrations generated by a heuristic optimizer, namely PSO. Comparing the learned policy with heuristic-based trajectory optimization methods is therefore the most direct way to evaluate whether the proposed method can preserve decision quality while significantly improving online computational efficiency.

This study’s key innovations are as follows: (1) The influence of active signal interference on the detection capability of multi-radar systems is considered, making the proposed trajectory planning method more suitable for complex signal interference environments. (2) The proposed trajectory policy representation neural network architecture design is based on multi-semantic information fusion. The architecture uses the powerful nonlinear mapping capability and fast online inference characteristics of deep neural networks to address insufficient real-time solving efficiency in traditional heuristic algorithms. (3) This study proposes a supervised training approach that uses the trajectory planning method of traditional heuristic algorithms as the imitation target to train the trajectory policy neural network and enhance its fast convergence capability.

## 3. System Modeling and Problem Formulation

Consider a cooperative detection scenario of a multi-radar system in a two-dimensional plane. The scenario is modeled in a two-dimensional horizontal plane for the following reasons. First, the UAV radars are assumed to operate at a fixed cruising altitude. This is a standard engineering practice in area-coverage missions where altitude regulation is handled independently by the flight control system. Second, the proposed method focuses on the horizontal deployment and heading control of the UAV radar network. The key decision variables, namely the horizontal positions and headings of the radars, lie entirely in the 2D plane. We believe the altitude dimension does not directly affect the coverage optimization objective formulated in this work and is therefore excluded from the trajectory planning model. Third, for targets flying at a known cruising altitude, such as bombers or jet fighters during the cruise phase, the altitude can be treated as a constant offset. Under this condition, the 2D horizontal coverage map is equivalent to the coverage cross-section at the target altitude in 3D space.

**Figure 1:**
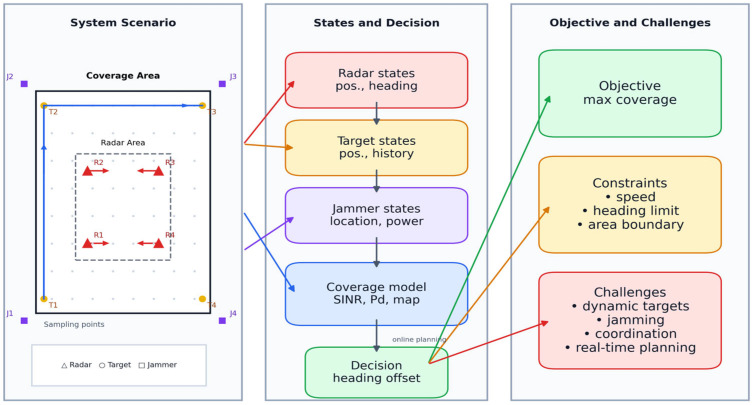
System model of multi-radar trajectory planning under signal interference.

The system model, illustrated in Figure 1, describes the components, state representation, and decision-making processes addressed in this study. Based on this framework, the subsequent sections present the mathematical formulation of the multi-radar trajectory planning problem, including the state definition, coverage modeling, and optimization objective.

The multi-radar system consists of N UAV radars spatially distributed over a wide area and capable of free maneuvering. The scenario also includes M target nodes and J fixed-interval support interference sources with noise suppression interference. The key coverage area is a rectangle with side lengths of $R_{x}\times R_{y}$, where $R_{x}$ and $R_{y}$ are the region side length along the x-axis and y-axis directions, respectively. The key coverage area is discretized into $L_{1}\times L_{2}$ sampling points in the two-dimensional plane, where $L_{1}$ and $L_{2}$ are the numbers of discrete sampling points along the x-axis and y-axis directions of the two-dimensional region, respectively. This point set Ω is denoted as

(1) $$\Omega =\left\{r_{1},\;r_{2},\ldots ,r_{L_{1}\times L_{2}}\right\},\;r_{l}\;\in \;R^{2},\;l=1,\ldots ,L_{1}\times L_{2},$$

Each UAV radar maneuvers within the restricted safe airspace $A_{r}$. The radars adjust its trajectory to achieve continuous coverage of the key area and effective detection of targets. The multi-radar system starts from time 0 and makes heading offset angle decisions at intervals of Δt to accomplish the tasks of key area coverage and target detection. For convenience, any discrete time instant $t\times \Delta t\;\left(t=0,1,\ldots T\right)$ is referred to simply as time t.

Let $P^{t}=\left(P_{1}^{t},\ldots ,P_{N}^{t}\right)\in {\mathbb{R}}^{2\times N}$ denote the position matrix of N UAV radars in the XOY horizontal plane at time t, where $P_{n}^{t}\in {\mathbb{R}}^{2\times 1}$ denotes the position of the n-th $\left(n=1,\ldots ,N\right)$ UAV radar; $\theta^{t}=\left(\theta_{1}^{t},\ldots ,\theta_{N}^{t}\right)\in {\mathbb{R}}^{1\times N}$ denotes the heading vector of N UAV radars at time t, where $\theta_{n}^{t}\in \mathbb{R}$ denotes the heading of the n-th $\left(n=1,\ldots ,N\right)$ UAV radar; $Q^{t}=\left(Q_{1}^{t},\ldots ,Q_{M}^{t}\right)\in {\mathbb{R}}^{2\times M}$ denotes the position matrix of M targets in the XOY horizontal plane at time t, where $Q_{m}^{t}\in {\mathbb{R}}^{2\times 1}$ denotes the position of the m-th $\left(m=1,\ldots ,M\right)$ target; $Z=\left(Z_{1},\ldots ,Z_{J}\right)\in {\mathbb{R}}^{2\times J}$ denotes the position matrix of J spatially separated interference sources in the XOY horizontal plane; and $Z_{j}$ denotes the position of the j-th $\left(j=1,\ldots ,J\right)$ interference source.

The trajectory decision problem of the multi-radar system can be described as computing the radar heading offset angle $\Delta \theta^{t}=\left(\Delta \theta_{1}^{t},\ldots ,\Delta \theta_{N}^{t}\right)\in {\mathbb{R}}^{1\times N}$. This problem is based on the positions of UAV radars, targets, and interference sources at time t, so as to compute the position of the UAV radar at time t+1; i.e.,

(2) $$P_{n}^{t+1}=P_{n}^{t}+v\Delta t\times \left[\begin{array}{c} \cos\left(\theta_{n}^{t+1}\right)\\ \sin\left(\theta_{n}^{t+1}\right) \end{array}\right],\;n=1,\ldots ,N,$$

(3) $$\theta_{n}^{t+1}=\theta_{n}^{t}+\Delta \theta_{n}^{t},n=1,\ldots ,N,$$

where v is the speed of the UAV, Δt is the time interval from time t to t+1, $\Delta \theta_{n}^{t}\in \left[-\Delta \theta_{max},\Delta \theta_{max}\right]$ denotes the heading offset angle of the n-th $\left(n=1,\ldots ,N\right)$ UAV radar at time t, and $\Delta \theta_{max}$ denotes the maximum offset angle within the time interval Δt.

## 4. Trajectory Planning Method Based on Imitation Learning

### 4.1. Trajectory Policy Representation Neural Network Architecture Based on Multi-Semantic Information Fusion

Although the proposed framework follows the general paradigm of expert demonstration and supervised policy approximation, its contribution is not limited to directly applying behavior cloning to a standard trajectory planning problem. Compared with existing imitation-learning or behavior-cloning studies in trajectory planning, this study introduces several task-specific advances. First, the considered problem is multi-radar cooperative trajectory planning in an active signal interference environment, which differs from conventional single-agent navigation or obstacle-avoidance settings. Second, a multi-semantic information fusion architecture is designed to jointly encode regional coverage information, single-radar coverage maps, radar maneuvering states, and target motion history, so that the learned policy is better aligned with the decision structure of the coverage-oriented radar planning task. Third, an auxiliary target-prediction task is incorporated during training to improve feature representation quality, accelerate convergence, and enhance generalization. The novelty of this work therefore lies not merely in adopting behavior cloning, but in developing a task-oriented imitation-learning framework for efficient online trajectory planning of multi-radar systems under signal interference conditions.

In the multi-radar trajectory planning task under signal interference environments, the multi-radar system needs to rapidly output multi-radar heading control commands based on the system state at time t. This state not only contains the maneuvering information of the radar itself, but is also simultaneously constrained by multiple factors such as regional coverage distribution, target motion trends, and signal interference effects, thus showing significant polysemy and heterogeneity. There are significant differences among different types of information regarding spatial structure, temporal correlation, and physical meaning. By taking a unified encoding approach, it will be difficult to fully extract the key features highly relevant to trajectory decision-making. To this end, this study designs a trajectory policy neural network architecture based on multi-semantic information fusion. The mathematical model of the trajectory policy network is expressed as

(4) $${\hat{\Delta \theta }}^{t}=f\left(s^{t};w\right),$$

where w is the neural network parameter, ${\hat{\Delta \theta }}^{t}\in {\mathbb{R}}^{N}$ is the heading offset angle output by the neural network, and $s^{t}=\left\{s_{all\_cov}^{t},s_{single\_cov}^{t},s_{\mathrm{uav}}^{t},s_{\mathrm{env}}^{t}\right\}$ is the multi-semantic information at time t, where $s_{all\_cov}^{t}\in {\left\{0,1\right\}}^{L_{1}\times L_{2}}$ denotes the multi-radar coverage information, $s_{single\_cov}^{t}\in {\left\{0,1\right\}}^{N\times L_{1}\times L_{2}}$ denotes the single-radar coverage information, $s_{\mathrm{uav}}^{t}=\left(\begin{array}{c} P^{t}\\ \theta^{t} \end{array}\right)\in {\mathbb{R}}^{3\times N}$ denotes the instantaneous state information of the multi-radar system, and $s_{\mathrm{env}}^{t}\in {\mathbb{R}}^{K\times 2\times M}$ denotes the target perception information of the multi-radar system over the preceding K time instants, i.e.,

(5) $$s_{env}^{t}=\left(\begin{array}{c} Q^{t}\\ \begin{array}{c} Q^{t-1}\\ \vdots \\ Q^{t-K+1} \end{array} \end{array}\right).$$

#### 4.1.1. Computation of Multi-Semantic Information

At each time instant t, the multi-semantic information $s^{t}$ serving as input to the neural network needs to be dynamically computed. Among these, $s_{\mathrm{uav}}^{t}$ and $s_{\mathrm{env}}^{t}$ are related to the radar’s own motion state information and target detection results. They are relatively straightforward to obtain. The following focuses on the construction methods for $s_{single\_cov}^{t}$ and $s_{all\_cov}^{t}$.

Let $P_{d,n}^{t}\left(r_{l}\right)$ denote the detection probability of the n-th $\left(n=1,\ldots ,N\right)$ UAV radar for a target at spatial position $r_{l}\;\left(l=1,\ldots ,L_{1}\times L_{2}\right)$. Computed according to constant false alarm rate (CFAR) detection theory, $P_{d,n}^{t}\left(r_{l}\right)$ can be expressed as

(6) $$P_{d,n}^{t}\left(r_{l}\right)\;\approx \;\frac{1}{2}erfc\left(\sqrt{-lnP_{fa}\;}-\sqrt{SNR_{n,l}^{t}}\right),$$

where $P_{fa}$ is the false alarm rate, and erfc is the complementary error function, i.e.,

(7) $$erfc\left(u\right)=1-\frac{2}{\sqrt{\pi }}\int_{0}^{u}e^{-v^{2}}dv,$$

and $SNR_{n,l}^{t}$ denotes the signal-to-interference-plus-noise ratio of the target echo at position $r_{l}$ received by radar n, i.e.,

(8) $$SNR_{n,l}^{t}=10log\frac{P_{n,l}}{\sigma_{w}^{2}+\sum_{J=1}^{J}P_{n,j}},$$

where $\sigma_{w}^{2}$ is the noise power, $P_{n,l}$ is the target echo power at position $r_{l}$ received by radar n, and $P_{n,j}$ is the interference power from the j-th stand-off support interference source received by radar n.

The coverage $s_{single\_cov}^{t}\left[n,l_{1},l_{2}\right]$ of the n-th $\left(n=1,\ldots ,N\right)$ UAV radar at spatial position $r_{l}$ is expressed as

(9) $$s_{single\_cov}^{t}\left[n,l_{1},l_{2}\right]=\left\{\begin{array}{c} 1,\;if\;P_{d,n}^{t}\left(r_{l}\right)\geq 0.5\\ 0,\;if\;\;P_{d,n}^{t}\left(r_{l}\right)<0.5 \end{array}\right.,\;l=l_{1}\times l_{2}.$$

The threshold of 0.5 follows the standard Neyman–Pearson detection criterion. This means the probability of detection and missed detection are equal. This value represents the minimum acceptable detection reliability in practice and provides a balanced, widely adopted coverage criterion in radar system design.

Assuming the multi-radar system adopts a multi-radar cooperative detection mechanism, the joint detection probability for a target at sampling point $r_{l}$ can be expressed as

(10) $$P_{d}^{t}\left(r_{l}\right)=1-\overset{N}{\underset{n=1}{\prod }}\left(1-P_{d,n}^{t}\left(r_{l}\right)\right),$$

and the coverage $s_{all\_cov}^{t}\left[l_{1},l_{2}\right]$ of the multi-radar system at spatial position $r_{l}$ is expressed as

(11) $$s_{all\_cov}^{t}\left[l_{1},l_{2}\right]=\left\{\begin{array}{c} 1,\;if\;\;P_{d}^{t}\left(r_{l}\right)\geq 0.5\\ 0,\;if\;\;P_{d}^{t}\left(r_{l}\right)<0.5 \end{array}\right.,\;l=l_{1}\times l_{2}.$$

#### 4.1.2. Multi-Semantic Information Feature Fusion

**Figure 2:**
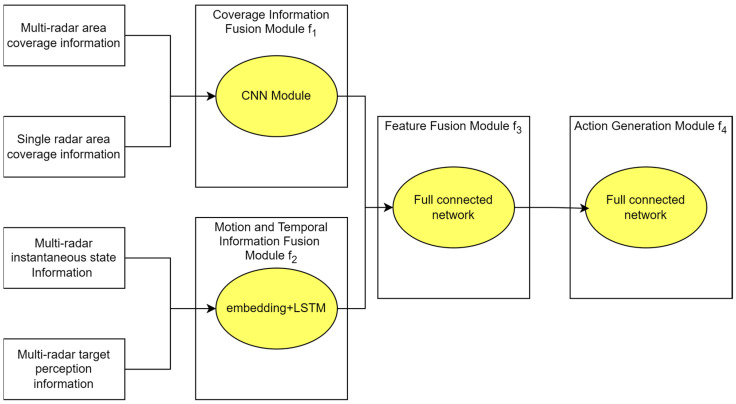
Architecture of the trajectory policy network based on multi-semantic information fusion.

The policy network architecture is shown in Figure 2. The trajectory policy neural network consists of a coverage information fusion module, a motion and temporal information fusion module, and a feature fusion module. The coverage information fusion module and the motion and temporal information fusion module perform fusion on different types of state information, respectively. After fusion, the results are uniformly input into the feature fusion module, which ultimately outputs the heading offset angle control commands for the multi-radar system.

The roles of the different modules of the policy network are as follows:

1. Coverage information fusion module $f_{1}$: produces output $t_{1}\in {\mathbb{R}}^{128}$ by performing convolutional operations on $s_{all\_cov}^{t}$ and $s_{single\_cov}^{t}$

(12) $$t_{1}=f_{1}\left(s_{all\_cov}^{t},\;s_{single\_cov}^{t};\;w_{1}\right),\;$$

2. Motion and temporal information fusion module $f_{2}$: produces output $t_{2}\in {\mathbb{R}}^{128}$ by performing embedding and long short-term memory (LSTM) operations on $s_{\mathrm{uav}}^{t}$ and $s_{\mathrm{env}}^{t}$

(13) $$t_{2}=f_{2}\left(s_{\mathrm{uav}}^{t},s_{\mathrm{env}}^{t};w_{2}\right),$$

3. Feature fusion module $f_{3}$: fuses the outputs $t_{1}$ and $t_{2}$ of the preceding two modules to obtain the fused feature vector $t_{3}\in {\mathbb{R}}^{256}$

(14) $$t_{3}=f_{3}\left(t_{1},t_{2};w_{3}\right),$$

4. Action generation module $f_{4}$: subsequently applies fully connected layer computation to $t_{3}$, and uses the tanh activation function to constrain the output values to the interval $\left[-1,\;1\right]$. It is then multiplied by the preset maximum offset angle $\Delta \theta_{\max}$. The generated N-dimensional action vector ${\hat{\Delta \theta }}^{t}$ is thus constrained within the permissible range. The final heading deflection angle ${\left[{\hat{\Delta \theta }}_{1}^{t},{\hat{\Delta \theta }}_{2}^{t},\ldots ,{\hat{\Delta \theta }}_{N}^{t}\right]}^{T}$ assigned to each radar is thus determined.

(15) $${\hat{\Delta \theta }}^{t}=f_{4}\left(t_{3};\;w_{4}\right),$$

### 4.2. Trajectory Policy Network Training Based on Imitation Learning

To address the problem of fast training of the trajectory policy network, this study proposes using the trajectory planning method of traditional heuristic algorithms as the imitation target. In addition, it employs a supervised approach to train the trajectory policy neural network.

The trajectory planning method of traditional heuristic algorithms generally solves the following mathematical model, i.e.,

(16)  max Ct+1 s.t.−Δθmax≤Δθnt≤Δθmax, n=1,…,N, t=0,…,T−1Pnt+1∈Ar, n=1,…,N, t=0,…,T−1Pnt+1=Pnt+vΔt∗cosθnt+Δθntsinθnt+Δθnt, n=1,…,N, t=0…,T−1,

where $C^{t+1}$ denotes the effective coverage rate of the key area at time t+1, i.e.,

(17) $$C^{t+1}=\frac{1}{L_{1}\times L_{2}}\sum_{l=1}^{L_{1}\times L_{2}}I\left(P_{d}^{t+1}\left(r_{l}\right)\geq 0.5\right),\;$$

where $I\left(\cdot \right)$ is the indicator function. Let $\Delta {\overset{ˇ}{\theta }}^{t}$ be the optimal solution of the above mathematical model. The imitation-learning training dataset is then defined as $X=\left\{\left(s^{t},\Delta {\overset{ˇ}{\theta }}^{t}\right)\right\}$, where t is a discrete sampling time point.

Based on the above dataset, training the policy network parameters is achieved by optimizing the following loss function:

(18) $$L_{BC}\left(w\right)\;=\;\sum_{t}{\left\|f\left(s^{t};w\right)-\Delta {\overset{ˇ}{\theta }}^{t}\right\|}^{2},\;$$

where $f\left(s^{t};w\right)$ denotes the heading offset angle output by the network, and $\Delta {\overset{ˇ}{\theta }}^{t}$ is the corresponding optimal heading offset angle obtained from the optimization model. The full primary task training objective, incorporating an $L_{2}$ regularization term, is:

(19) $$L\left(w\right)\;=\;L_{BC}\left(w\right)\;+\;\lambda \|w\|,$$

where λ>0 is a regularization hyperparameter. Here, λ denotes the regularization hyperparameter associated with the $L_{2}$ penalty term on the network parameters. Its role is to prevent overfitting by discouraging excessively large parameter values and thereby improving the generalization capability and training stability of the trajectory policy network. In practice, λ controls the trade-off between fitting the expert demonstration data and keeping the model complexity moderate. A too small value may lead to overfitting, whereas an excessively large value may cause underfitting and degrade action imitation accuracy. In this work, λ is selected empirically based on training stability and validation performance.

To further improve the convergence speed and generalization performance of the trajectory policy network, an auxiliary task is introduced only during the training phase. Specifically, an additional prediction head $f_{5}$ is appended after the feature fusion module output $t_{3}$. This module takes $t_{3}$ as input and outputs the predicted target positions ${\hat{Q}}^{t}=f_{5\;}\left(t_{3};w_{5}\right)\in {\mathbb{R}}^{2\times M}$ at time t. To decrease the influence of positional scale differences on the loss magnitude, the coordinates of the predicted and ground-truth target positions are normalized by the maximum side length of the key coverage area. The auxiliary task loss is then defined as:


(20) $$L_{aux}\left(w\right)\;=\;\sum_{t}\;{\left|\frac{{\hat{Q}}^{t}}{max\left(R_{x},\;R_{y}\right)}-\frac{Q^{t}}{max\left(R_{x},\;R_{y}\right)}\right|}^{2}.$$

When the auxiliary task is enabled, the overall training loss becomes:

(21) $$L_{total}\left(w\right)\;=\;L_{BC}\left(w\right)\;+\;\alpha L_{aux}\left(w\right)\;+\;\lambda \|w\|,$$

where $\alpha \in \left\{0,1\right\}$ is an auxiliary task flag that enables the auxiliary tasks. It is important that the auxiliary prediction head and its associated loss $L_{aux}$ serve solely as a training-phase regularization to guide feature representation learning. During inference, the auxiliary head is removed, and the network outputs only the heading offset angles. To ensure a fair comparison between the configurations with and without the auxiliary task, the primary task loss $L\left(w\right)=L_{BC}\left(w\right)+\lambda \|w\|$ defined in Equation (19) is used as the unified evaluation metric in all experiments, regardless of whether the auxiliary task is incorporated during training.

The overall procedure of the imitation training algorithm is presented as Algorithm 1: **Algorithm 1**. Imitation Training Algorithm for Multi-Radar Trajectory Decision-Making Based on Imitation Learning**Input:** Number of decision time instants T; particle swarm optimization solver $solver\left(\cdot \right)$; learning rate η; number of training epochs E; batch size B; loss function hyperparameter λ; auxiliary task flag $\alpha \in \left\{0,1\right\}$.
**Output:** Trajectory policy network parameters w.
**Step 1: Training Data Generation**
Initialize training data X←∅;**for** t=0 **to** T−1 **do**Establish the optimization model shown in (16);Update $s^{t}\leftarrow \left\{s_{all\_cov}^{t},s_{single\_cov}^{t},\;s_{\mathrm{uav}}^{t},\;s_{\mathrm{env}}^{t}\right\}$;Call the particle swarm optimization solver to obtain $\Delta {\overset{ˇ}{\theta }}^{t}=\left(\Delta {\overset{ˇ}{\theta }}_{1}^{t},\ldots ,\Delta {\overset{ˇ}{\theta }}_{N}^{t}\right)\leftarrow \underset{\Delta {\overset{ˇ}{\theta }}_{n}^{t}}{\underset{︸}{argmax}}\left\{\mathrm{model}\left(16\right)\right\},n=1,\ldots ,N$Update training dataset $X\leftarrow X\cup \left\{\left(s^{t},\Delta {\overset{ˇ}{\theta }}^{t}\right)\right\}$;Execute action $\Delta {\overset{ˇ}{\theta }}^{t}=\left(\Delta {\overset{ˇ}{\theta }}_{1}^{t},\ldots ,\Delta {\overset{ˇ}{\theta }}_{N}^{t}\right)$, update the positions and headings of the multi-radar system according to equations $\theta_{n}^{t+1}=\theta_{n}^{t}+\Delta {\overset{ˇ}{\theta }}_{n}^{t},P_{n}^{t+1}=P_{n}^{t}+v\Delta t*\left[\begin{array}{c} \cos\left(\theta_{n}^{t+1}\right)\\ \sin\left(\theta_{n}^{t+1}\right) \end{array}\right],n=1,\ldots ,N$;t←t+1;**end for****return** X
**Step 2: Updating Trajectory Policy Network Parameters via Imitation Learning**
Randomly initialize trajectory policy network parameters w;**for** epoch=1 **to** E **do**Sample a batch from dataset X: ${\left\{\left(s^{i},\Delta {\overset{ˇ}{\theta }}^{i}\right)\right\}}_{i=1}^{B}$;Compute loss function $L\left(w\right)\leftarrow \frac{1}{B}\sum_{i=1}^{B}{\left\|f\left(s^{i};w\right)-\Delta {\overset{ˇ}{\theta }}^{i}\right\|}^{2}+\alpha L_{aux}\left(w\right)+\lambda \|w\|$;Backpropagate to compute gradient $g\leftarrow \nabla_{w}L\left(w\right)$;Update parameters w←w−η·g;**end for****return** w

## 5. Simulation Experiments

### 5.1. Implementation Details

The policy network comprises two convolutional branches for processing the joint detection probability map $s_{all\_cov}^{t}$ and the per-radar detection maps $s_{single\_cov}^{t}$, each comprising two convolutional layers with 32 and 64 channels, respectively (kernel size 3 × 3, padding 1), followed by adaptive average pooling and flattening. The radar state $s_{\mathrm{uav}}^{t}$ is embedded via a fully connected layer (12→64), and the target history $s_{\mathrm{env}}^{t}$ is processed by a single-layer LSTM with hidden dimension 64, preceded by a linear embedding layer (8→64). The four feature vectors are concatenated into a 256-dimensional joint representation, which is then passed through a four-layer MLP (256→512→256→128→4) to produce the action output. The final layer is initialized with orthogonal initialization (gain = 0.01) to stabilize early training.

The network is trained using the Adam optimizer [25] (PyTorch v2.6.0; Meta Platforms, Inc., Menlo Park, CA, USA) with a learning rate of ${3\times 10}^{-4}$ and weight decay of $10^{-4}$. The batch size is set to 64 and training runs for 1000 epochs. Gradient clipping with a maximum norm of 1.0 is applied to prevent gradient explosion. The auxiliary task weight is set to α=0.3 based on the ablation study presented in Section 5.4. All experiments are conducted on a platform equipped with an AMD Ryzen 5 7500F CPU and an NVIDIA RTX 3090 GPU. The experimental environment is Python version 3.12.

### 5.2. Experimental Scenario Description

**Figure 3:**
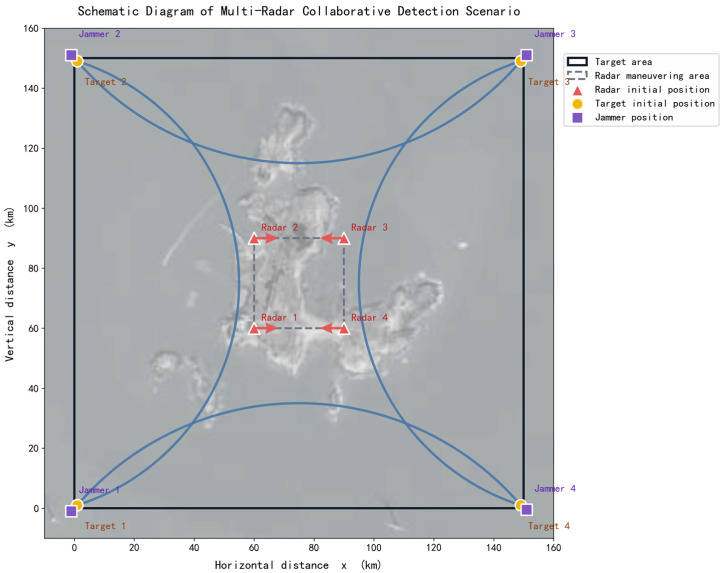
Simulation scenario showing radars, targets, and interference sources in the coverage area.

As shown in Figure 3, yellow circles represent targets, red triangles represent radars, and purple squares represent interference sources. The targets attempt to traverse the surveillance coverage area along the blue arc trajectory. The radar nodes are each equipped with a three-face radar and move within the movable area bounded by the dashed rectangle. They attempt to maximize the coverage of the entire rectangular area to be covered and to track and detect the targets.

For simplicity, the simulation scenario is represented in a Cartesian coordinate system with the horizontal and vertical axes denoting range in kilometers. Earth curvature and coordinate projection effects are neglected, which is a standard simplification for localized airborne radar network scenarios covering areas on the order of 150 km × 150 km.

In the simulation scenario, the target movement trajectory is arc-shaped, the blue arc represents the flight path of the target. The variation in target position with time can be expressed by the following equation:

(22) $$\left\{\begin{array}{c} \varphi_{m}^{t}\;=\;\varphi_{m}^{0}\;+\;\Delta t\cdot \phi \\ x_{m}^{t}\;=\;x_{m}^{0}\;+\;r\cdot cos\left(\varphi_{m}^{t}\right)\\ y_{m}^{t}\;=\;y_{m}^{0}\;+\;r\cdot sin\left(\varphi_{m}^{t}\right) \end{array}\right.,$$

where r and ϕ are the radius and angular velocity of the arc trajectory, taking values of 81.25 km and 4.49°/s, respectively.

The simulation experiment parameters are presented in Table 1. The initial radar information, initial target information, and interference source information in the simulation scenario are provided in Table 2, Table 3, and Table 4, respectively.

The radar and interference source parameter settings used in the simulation are summarized in Table 1. Among them, the radar transmit power is determined from the assumed detection-range requirement, while the interference source power is selected as a representative scenario parameter for contested-environment evaluation. The main radar parameters are specified as follows: the operating frequency is 75 MHz, the signal bandwidth is 10 MHz, the transmit and receive antenna gains are both 30 dB, the system loss is 12.5 dB, and the target radar cross section is 16 m^2^. The expected detection range is set to 90 km. Based on these assumptions, the required radar transmit power is calculated to be 4500 W. The radar transmit power used in the simulation is therefore not an arbitrary choice, but a value determined from the assumed detection-performance requirement. The interference source transmit power is set to 1000 W as a scenario-setting parameter. This value is used to construct a challenging signal interference scenario in which the interference source produces significant suppression effects while the radar system still retains a certain level of detectability. In this way, the robustness and trajectory adaptation capability of the proposed method can be meaningfully evaluated. The interference source power should therefore be understood as a representative simulation setting rather than the specification of a particular interference source product.

### 5.3. Training Performance Experiment

**Figure 4:**
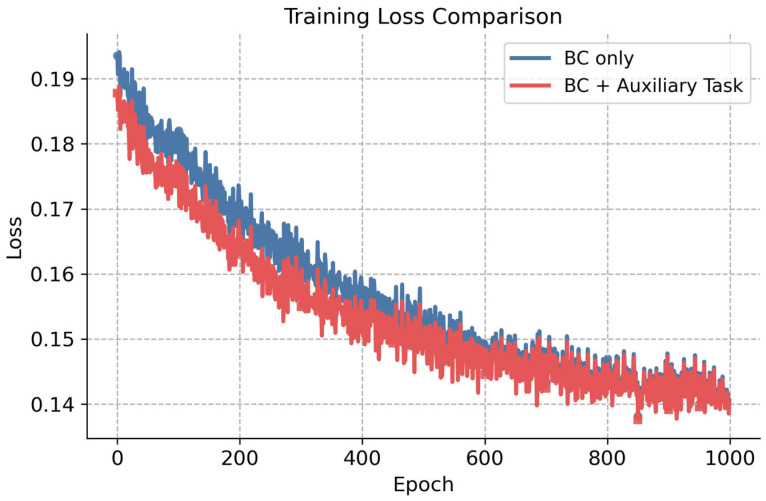
Variation in the policy network training loss over epochs.

In this study, the particle swarm optimization algorithm is used to solve the single-step trajectory optimization mathematical model. The solutions obtained by this algorithm are used as the training data for the trajectory policy network. Figure 4 illustrates the variation in the primary task loss function $L\left(w\right)$ during the training process under the configurations with and without the auxiliary task. As shown in the figure, both curves exhibit an overall downward trend, decreasing from 0.19 to 0.14. The key difference is that the configuration with the auxiliary task converges more rapidly during the first 400 epochs compared to the configuration without it. The two curves gradually converge after 600 epochs. This shows that incorporating the auxiliary task can accelerate the convergence of the primary task during training.

**Figure 5:**
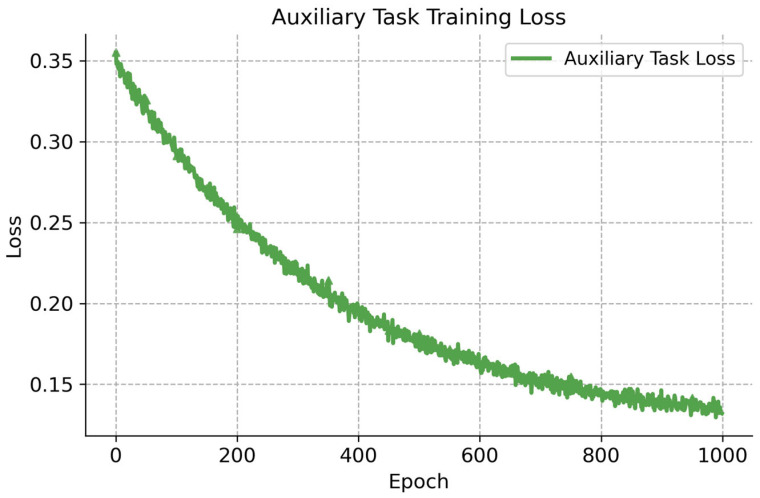
Training loss of the auxiliary task over epochs.

Figure 5 presents the variation in the auxiliary task loss $L_{aux}$ during the training process. The auxiliary task loss exhibits a consistent downward trend, decreasing from 0.35 to 0.13 over 1000 epochs. This indicates that the auxiliary task converges stably and achieves satisfactory performance in target position prediction.

**Figure 6:**
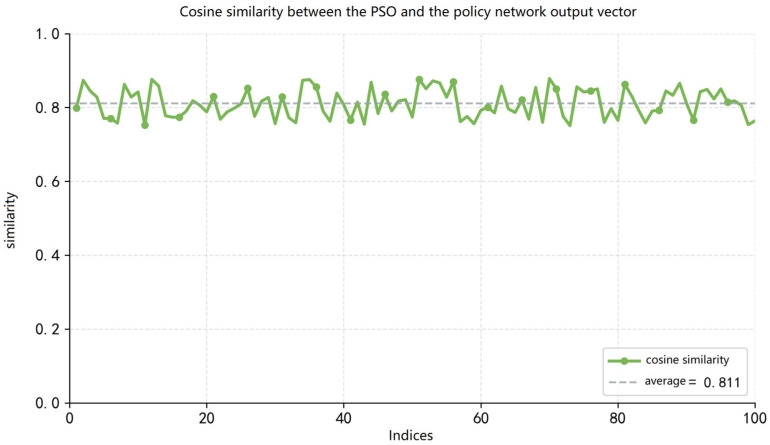
Similarity between actions generated by the trajectory policy network and PSO on random samples.

Figure 6 shows the similarity between the output of the trajectory policy network and the particle swarm method on random samples. The average similarity across 100 samples can reach 81.1%, as indicated by the gray dashed line in the figure. The lowest similarity of 75.2% appears in the 91st sample, while the highest similarity of 88.0% is achieved in the 53rd sample. It shows the good imitation performance of the policy network.

**Figure 7:**
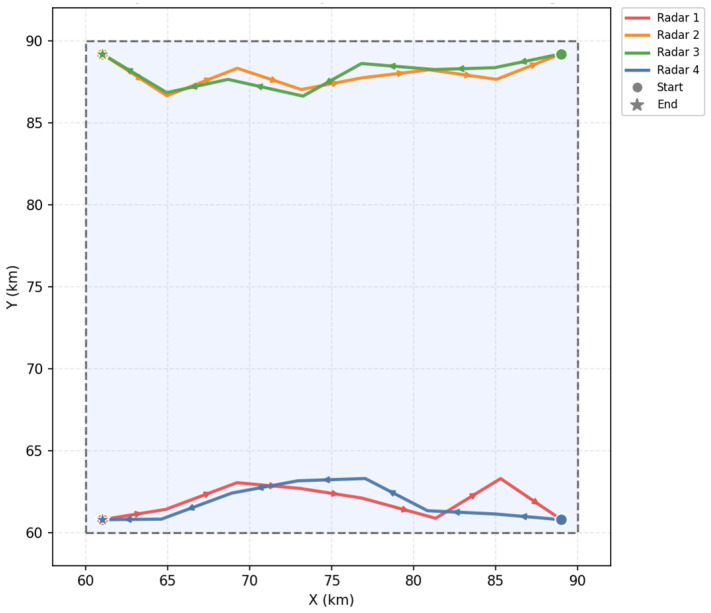
Example of radar trajectories within the maneuvering area.

As shown in Figure 7, the radars move near the boundary of the maneuvering area while performing slight inward adjustments. The dashed lines are the areas of radar activity. Lines of different colors represent the tracks of each radar. The dots above represent the position of each radar at different times. This enables them to remain relatively closer to the target region and to better maintain cooperative coverage over the whole area, which is beneficial for improving the overall coverage rate. This behavior reflects the tendency of the radars to adjust their positions within the feasible region while preserving overall directional consistency.

### 5.4. Auxiliary Task Ablation

**Figure 8:**
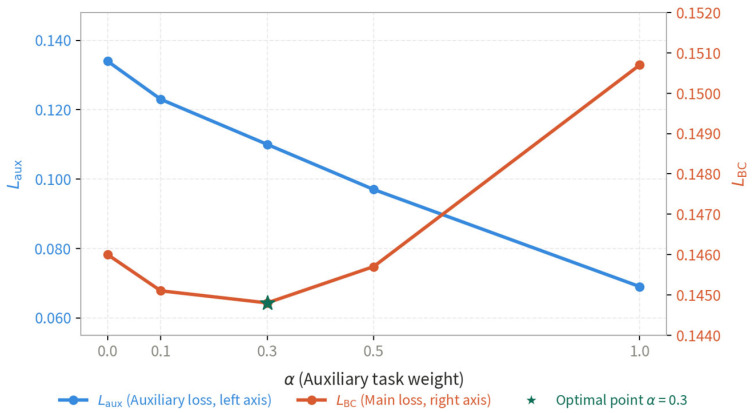
Effect of auxiliary task weight α on training loss.

To further investigate the effect of the auxiliary task weight α, we train the policy network under five different α values: {0, 0.1, 0.3, 0.5, 1.0}. As shown in Figure 8, the auxiliary loss $L_{\mathrm{aux}}$ decreases monotonically with increasing α, confirming that a larger auxiliary weight drives the network to learn more accurate target position representations. Meanwhile, the primary task loss $L_{\mathrm{BC}}$ exhibits a U-shaped curve with a minimum at α=0.3, indicating that an intermediate auxiliary weight provides the most beneficial regularization effect on the main task. Specifically, α=0.3 achieves a 1.0% reduction in $L_{\mathrm{BC}}$ compared to the baseline without the auxiliary task (α=0), and a 4.3% reduction compared to the over-weighted setting (α=1.0). Based on this analysis, α=0.3 is adopted as the default configuration in all experiments.

### 5.5. Inference Capability Experiment

**Figure 9:**
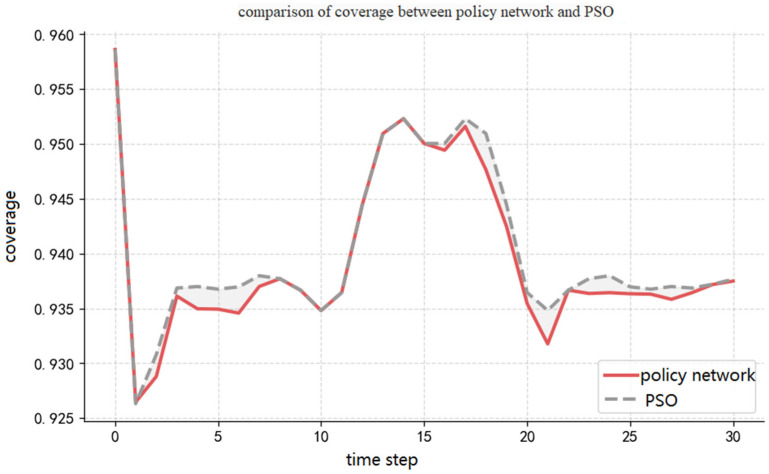
Comparison of coverage rate between the trajectory policy network and PSO over time.

Figure 9 describes the experimental results of the trajectory policy network proposed in this study and the particle swarm algorithm regarding coverage capability. In the figure, the gray dashed line represents the variation in the coverage rate of the particle swarm algorithm, and the red solid line represents the variation in the coverage rate of the imitation-learning method. During the time period t=0~1, the coverage rate of both methods decreases from 95.86% to 92.63%. This is because, at the initial moment, the radars, targets, and signal interference sources are all in a uniformly distributed state. As the radars and targets move forward, their motion is constrained by the radar’s own motion constraints and boundary constraints. The overall system coverage rate experiences a phased decline. Subsequently, during the time period t=2~13, the radars gradually adjust their positions and form an effective deployment. The coverage rate of both methods shows an upward trend, rising from 92.63% to 95.09%. During the time period t=14~18, the coverage rate stabilizes and fluctuates around 95.00%. After t=20, the coverage rate of both methods oscillates slightly around 93.77%.

From the overall trend, the trajectory policy network is basically consistent with the particle swarm algorithm regarding coverage rate variation characteristics. The policy network effectively reproduces the decision-making behavior of the particle swarm algorithm. However, regarding quantitative indicators, the trajectory policy network achieves an average coverage rate of 93.95% across all decision points throughout the entire process, which is lower than the particle swarm algorithm’s 94.02%. This difference mainly stems from the inevitable error accumulation effect of imitation-learning in sequential decision-making processes.

The slight performance gap between the policy network (93.95%) and PSO (94.02%) results from compounding errors inherent in behavior cloning: small deviations from the expert trajectory accumulate over successive time steps, driving the system into state regions underrepresented in training data. As shown in Figure 8, this gap is most pronounced after t > 20, confirming its sequential compounding nature. Incorporating online correction mechanisms such as DAgger is identified as a direction for future work.

**Figure 10:**
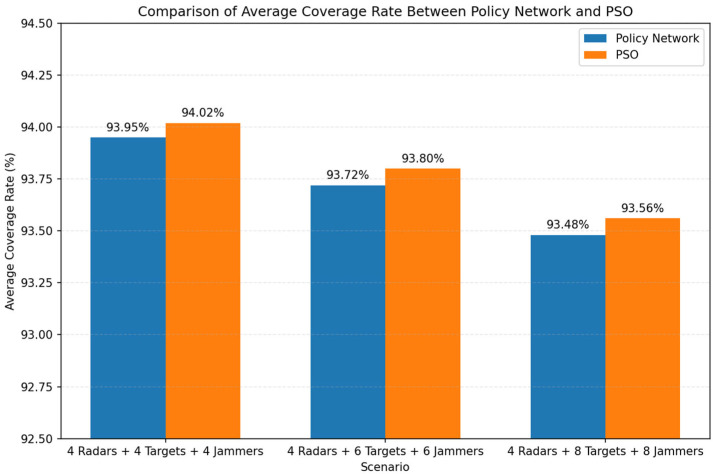
Average coverage rate under different radar–target configurations.

Figure 10 presents the comparison of average coverage rate between the proposed policy network and the PSO-based method under different radar–target–interference source configurations. The policy network achieves coverage performance that is consistently close to that of PSO across all scenarios. Specifically, in the case of 4 radars and 4 targets, the policy network attains an average coverage rate of 93.95%, which is slightly lower than the 94.02% achieved by PSO. Similar trends can be observed in more complex scenarios. When the number of targets increases to 6 and 8, the coverage rates of both methods decrease slightly, with the policy network achieving 93.72% and 93.48%, compared to 93.80% and 93.56% for PSO, respectively.

From the overall results, two observations can be made. First, the proposed method is able to closely approximate the performance of the PSO-based heuristic optimizer, with only marginal degradation (less than 0.1% in all cases). This indicates that the imitation-learning framework effectively captures the decision behavior of the expert solver. Second, as the scenario complexity increases (i.e., more targets and interference sources), the coverage performance of both methods decreases slightly, which is expected due to the increased competition for coverage resources. However, the relative performance gap between the two methods remains stable, demonstrating the robustness of the learned policy. These results confirm that the proposed policy network can maintain high coverage performance while significantly reducing computational cost, making it a practical alternative to iterative heuristic optimization methods.

**Figure 11:**
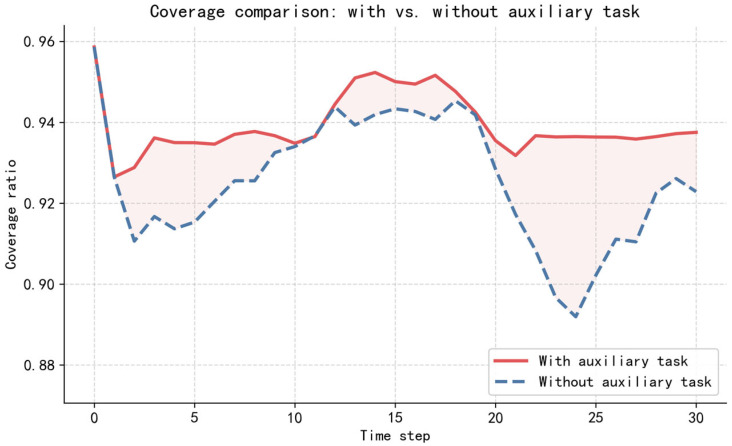
Comparison of coverage performance between policy networks trained with and without the auxiliary task.

Figure 11 presents a comparison of the coverage rate between the policy network trained with the auxiliary task and that trained without it. From the overall trend, the policy network trained with the auxiliary task consistently achieves a higher coverage rate than that trained without it throughout the entire decision-making process. Regarding the temporal performance, the coverage rate of the configuration with the auxiliary task is described in Figure 8. The configuration without auxiliary tasks experiences a sharp drop in coverage from 95.86% to 91.03% at t=2, followed by a steady increase reaching 94.42% at t=12. From t=12 to t=19, the coverage rate fluctuates around 94%. It then begins to decline, reaching its lowest point of 89.27% at t=24. Coverage subsequently recovers to 92.31% in the subsequent steps. The policy network trained with the auxiliary task demonstrates superior coverage performance compared to that trained without it. This indicates that the auxiliary task yields a significant beneficial effect during the training of the trajectory policy network.

**Figure 12:**
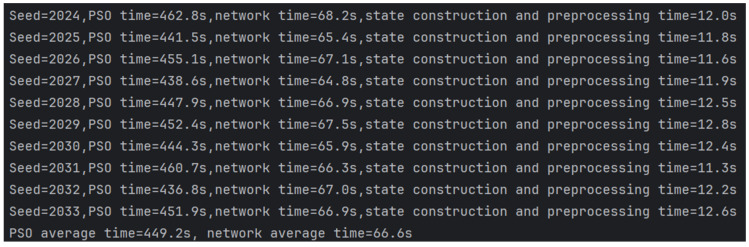
Running time comparison between PSO and the policy network under different random seeds.

**Figure 13:**
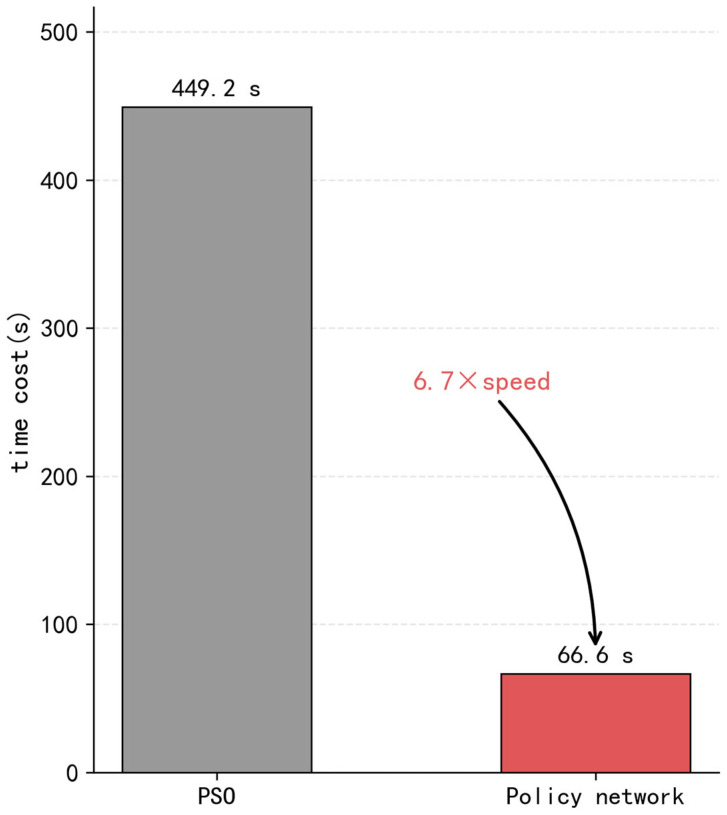
Comparison of computational time between the trajectory policy network and PSO.

Figure 12 reports the running times of PSO and the policy network under 10 different random seeds. For each seed, the running time of PSO is consistently much higher than that of the policy network. The average running time of PSO is 449.2 s, whereas the average running time of the policy network is 66.6 s, indicating that the proposed method achieves a substantial improvement in computational efficiency. In addition, the reported policy-network time includes the time required for state construction and inference preprocessing, which is approximately 12 s in each run. Figure 13 describes the experimental results of the trajectory policy network proposed in this study and the particle swarm algorithm regarding computational efficiency. The average computation time of the particle swarm algorithm is 449.19 s, while the trajectory policy network requires only 66.58 s. This result represents an improvement in computation speed of approximately 6.7 times that of the particle swarm algorithm. This result indicates that the proposed policy network has the potential to satisfy the real-time requirement of online trajectory decision-making in the considered multi-radar scenario. Since the policy network replaces iterative optimization with direct forward inference, the substantial reduction in computation time demonstrates its practical feasibility for real-time online trajectory planning under the considered simulation setting.

Beyond the average running times, the efficiency difference between the two methods can be explained by their distinct online decision mechanisms. For PSO, each decision step requires iterative population-based optimization, where multiple candidate actions must be repeatedly evaluated before selecting the final heading command. As a result, its online computational cost accumulates over iterations and becomes substantial. In contrast, the proposed policy network replaces iterative search with direct feed-forward inference after offline training. Its online running time mainly consists of state construction, preprocessing, and a single forward pass of the neural network. The online computational burden is therefore significantly reduced.

## 6. Discussion

This study proposes an imitation-learning-based trajectory planning method to solve the problem of insufficient real-time performance of multi-radar trajectory planning in complex electromagnetic signal interference environments. The study introduces an effective coverage rate metric for key areas based on detection probability. It constructs a performance evaluation model that comprehensively reflects the effects of radar deployment, trajectory maneuvering, and signal interference suppression. On this basis, a trajectory decision model targeting coverage rate improvement is designed, and expert demonstration data are generated using heuristic trajectory planning methods. The study employed imitation learning to perform supervised training of the neural network, achieving effective approximation of the expert trajectory decision strategy.

Simulation experimental results demonstrate that the proposed method can significantly reduce the computational complexity of online trajectory decision-making while maintaining high coverage performance. It achieves fast and stable heading control output that satisfies the real-time trajectory planning requirements of multi-radar systems in complex electromagnetic environments. This method avoids the drawbacks of traditional methods such as high computational complexity and long computation time. It also avoids the problems of long training time and difficulty in convergence associated with training neural networks for decision-making from scratch. This method achieves a combination of the two approaches.

PSO is selected as the comparison baseline for two reasons. First, heuristic algorithms are the predominant solver class for multi-radar trajectory planning in the current literature. Second, the proposed method is explicitly trained on PSO-generated expert demonstrations, making a direct comparison with PSO the most appropriate way to quantify the approximation quality and computational gain achieved by the learned policy.

## 7. Conclusions

This study proposes an imitation-learning-based trajectory planning method for multi-radar systems operating in complex electromagnetic signal interference environments. To address the limitations of traditional heuristic methods regarding computational complexity and real-time performance, a trajectory policy neural network architecture based on multi-semantic information fusion is designed. The architecture integrates regional coverage status, target motion information, and radar maneuvering state through a dedicated coverage information fusion module and a motion-temporal information fusion module, enabling rich heterogeneous state representations. A training dataset is constructed offline using the particle swarm optimization algorithm as the expert demonstrator, generating state–action pairs that encode near-optimal single-step heading decisions. An imitation-learning training strategy incorporating an auxiliary target prediction task is further proposed to accelerate convergence and enhance generalization.

Simulation results demonstrate that the proposed method achieves an average coverage rate of 93.95%, closely approximating the 94.02% attained by the PSO solver, while reducing the single-step decision time from 449.19 s to 66.58 s, representing a 6.7-fold improvement in computational efficiency. The auxiliary task is shown to accelerate primary task convergence during the early training phase without degrading final coverage performance. These results confirm that the proposed method effectively balances coverage quality and real-time decision-making capability, making it well-suited for deployment in dynamic and interfering electromagnetic environments.

Several directions remain open for future investigation. First, the current framework is validated under a fixed scenario configuration with four radars, four targets, and four interference sources in a two-dimensional plane. Extending the method to three-dimensional operational spaces and varying numbers of agents would improve its generalizability. Second, the behavior cloning paradigm used here is susceptible to compounding errors in sequential decision-making; incorporating online correction mechanisms such as DAgger or hybrid reinforcement–imitation learning could mitigate this issue. Third, this research focuses primarily on coverage rate as the optimization objective; incorporating additional task-level metrics such as continuous target tracking quality, interference robustness, and inter-radar cooperative balance would yield a more comprehensive performance evaluation. Finally, hardware-in-the-loop validation on physical radar platforms constitutes an important next step toward practical deployment.

## Figures and Tables

**Table 1:** Experiment parameters.

Parameters	Value
Number of radar nodes N	4
Number of target nodes M	4
Number of interference source nodes J	4
Number of decision time instants T	30
Coverage area range	(150 km, 150 km)
Radar antenna gain	30 dB
Interference source antenna gain	30 dB
Radar flight speed	200 m/s
Target radar cross section	16 m^2^
Movable area range	(30 km, 30 km)
Number of preceding time instants K	5
Radar transmit power	4500 W
Stand-off support interference source transmit power	1000 W
Maximum radar heading deflection angle	30°

**Table 2:** Radar initial information.

Radar Initial Position and Heading			
Radar 1	Radar 2	Radar 3	Radar 4
(60 km, 60 km, 0°)	(60 km, 90 km, 0°)	(90 km, 90 km, 180°)	(90 km, 60 km, 180°)

**Table 3:** Target initial information.

Target Initial Position			
Target 1	Target 2	Target 3	Target 4
(0 km, 0 km)	(0 km, 150 km)	(150 km, 150 km)	(150 km, 0 km)

**Table 4:** Interference source information.

Interference Source Position			
Interference source 1	Interference source 2	Interference source 3	Interference source 4
(0 km, 0 km)	(0 km, 150 km)	(150 km, 150 km)	(150 km, 0 km)

## Data Availability

The data supporting the findings of this study are not publicly available due to confidentiality restrictions.

## Abbreviations

The following abbreviations are used in this manuscript:

UAV	Unmanned Aerial Vehicle
PSO	Particle Swarm Optimization
CFAR	Constant False Alarm Rate
SNR	Signal-to-Noise Ratio
SINR	Signal-to-Interference-plus-Noise Ratio
DQN	Deep Q-Network
DDPG	Deep Deterministic Policy Gradient
PPO	Proximal Policy Optimization
MADDPG	Multi-Agent Deep Deterministic Policy Gradient
CNN	Convolutional Neural Network
LSTM	Long Short-Term Memory
BC	Behavioral Cloning
